# Outcomes, Data, and Indicators of Violence at the Community Level

**DOI:** 10.1007/s10935-016-0429-4

**Published:** 2016-03-11

**Authors:** Saba W. Masho, Michael E. Schoeny, Daniel Webster, Eric Sigel

**Affiliations:** Division of Epidemiology, Department of Family Medicine and Population Health, Virginia Commonwealth University, P.O. Box 980212, Richmond, VA 23298 USA; University of Chicago, Chicago, IL USA; Johns Hopkins University, Baltimore, MD USA; University of Colorado at Denver, Denver, CO USA

**Keywords:** Youth violence, Surveillance, Violence, Community level outcomes

## Abstract

Youth violence is a major problem in the United States. It remains the third leading cause of death among youth between the ages of 10 and 24 years and the leading cause of death in Blacks between 10 and 24 years of age. In its effort to prevent youth violence, the Center for Disease Control and Prevention funds six Youth Violence Prevention Centers (YVPCs) to design, implement and evaluate community-based youth violence prevention programs. These Centers rely on surveillance data to monitor youth violence and evaluate the impact of their interventions. In public health, surveillance entails a systematic collection and analysis of data, typically within defined populations. In the case of youth violence, surveillance data may include archival records from medical examiners, death certificates, hospital discharges, emergency room visits, ambulance pickups, juvenile justice system intakes, police incident reports, and school disciplinary incidents and actions. This article illustrates the process the YVPCs used for collecting and utilizing youth violence surveillance data. Specifically, we will describe available surveillance data sources, describe community-level outcomes, illustrate effective utilization of the data, and discuss the benefits and limitations of each data source. Public health professionals should utilize local surveillance data to monitor and describe youth violence in the community. Further, the data can be used to evaluate the impact of interventions in improving community-level outcomes.

## Introduction

Violence is among the most serious threats to the health and safety of youth in the United States (Mercy, Butchart, Farrington, & Cerdá, 2002). In 2011, homicide was the third leading cause of death in the United States among individuals aged 10–24 years and the leading cause of death for Blacks aged 10–24 years of age (CDC, 2014a, b). With over 4700 homicides in youth aged 10–24 in 2011, nearly 13 youth were killed every day. Homicide and assault-related injuries cost the United States over $17.5 billion USD in medical and work-related costs (CDC, 2014c).

Fatalities represent the most severe consequences of youth violence. However, nonfatal injuries are much more widespread, wrecking the wellbeing of communities. About 634,000 youth aged 10–24 years—approximately one in every 1000 youth—were treated in hospital emergency departments in 2012 for injuries related to interpersonal violence (CDC, 2014d). Disability, loss of productivity, increased burden on health and welfare services, and neighborhood decay are some of the well-known societal sequelae of violence (Mercy et al., 2002). Due to its severe consequences, the Department of Health and Human Services set reductions in violence-related morbidity and mortality as a high public health priority in the Healthy People 2020 goals (U.S. Department of Health and Human Services, 2013). As part of its effort, the Division of Violence Prevention at the Center for Disease Control and Prevention (CDC) has established several programs and initiatives that include surveillance to monitor trends of youth violence, and research to identify risk and protective factors associated with violence and to guide public health interventions and policies (Vivolo, Matjasko, & Massetti, 2011).

For over a decade, the CDC has partnered with a number of higher education institutions to create Youth Violence Prevention Centers (YVPCs; Vivolo et al., 2011). These institutions are equipped with scientists and programs to advance youth violence prevention initiatives. Currently, the YVPCs include the Johns Hopkins University (JHU), the University of Chicago (UC)/University of Illinois at Chicago (UIC), University of Colorado Boulder (CU-B), University of Michigan (UM), University of North Carolina at Chapel Hill (UNC), and Virginia Commonwealth University (VCU). These universities work with communities that are affected by high levels of youth violence. Through collaboration with local agencies, the YVPCs seek to implement and evaluate a set of strategies to improve violence prevention within their respective communities. The YVPCs have unique partnerships and expertise in utilizing surveillance data to monitor youth violence, evaluate prevention programs, and inform policies and future research activities (CDC, 2014f, g, h; Masho, Bishop, Edmonds, & Farrell, 2014). This article discusses experiences learned from these YVPCs using surveillance data to examine community-wide outcome indicators.

Public health surveillance data lend themselves to measure youth violence community outcomes. Public health surveillance involves the systematic collection, management, analysis, and interpretation of data for dissemination and public health action (Porta, 2008). Youth violence surveillance entails the collection and utilization of existing data from several sources. Archival data play a critical role in youth violence research, planning, monitoring, evaluation and policy formulation. Surveillance may be active, passive, or sentinel. Active surveillance involves direct monitoring of youth violence, and is often initiated by a local agency (such as a school system or health department) interested in the utility of the data. The CDC also actively collects data via nationally representative surveys such as the Youth Risk Behavior Surveillance System (YRBSS) to monitor the prevalence of youth violence and other adolescent health-risk behaviors in collaboration with the states (CDC, 2014e).

Data collected via passive surveillance, for example crime incident reports, are often reported by service-providing sectors or healthcare providers without direct action from potentially interested agencies. This information, while less generalizable, is less expensive to collect, easy to access and enables prompt and flexible monitoring and investigation of a specific problem. Sentinel surveillance, like active surveillance, is useful when higher-quality data are needed. Sentinel surveillance systems, such as the CDC’s National Violent Death Reporting System, entails a systematic identification of selected reporting sources who agree to report all cases of a defined problem (Birkhead & Maylahn, 2000). However, sentinel surveillance is restricted to selected areas of interest and is not commonly used in youth violence surveillance (WHO, 2014). Overall, the majority of surveillance data currently collected concerning youth violence is passively collected by service providing agencies. Public health agencies need community-level outcome indicators that can be uniformly used to monitor and evaluate programs (McDonald et al., 2012). Despite its availability, most communities do not utilize surveillance data to monitor the impact of violence or guide interventions. The CDC-funded YVPCs play a major role building academic-community partnerships that facilitate the utility of surveillance data to support community-wide efforts.

Current YVPC efforts are designed to have a community-wide impact on youth violence and are based on robust and representative community-level data (Griffith et al., 2008). This article identifies surveillance data sources that measure community-level indicators that are being used by the YVPCs. Specifically, we will list and describe available data sources, define community-level indicators for measuring youth violence, delineate challenges in analyzing the data and discuss effective utilization of surveillance data with specific examples, and discuss the strengths and weaknesses of each data source.

## Data Sources and Descriptions

This section describes local and national surveillance data sources that are used by the YVPCs and have the potential to be utilized by programs, researchers, and communities to understand fatal and nonfatal youth violence outcomes (Table 1). Further, we summarize community-level indicators used by the YVPCs for measuring youth violence and discuss the strengths and weaknesses of each data source.

**Table 1:** Youth violence surveillance data sources and characteristics

Data source	Description	Outcome indicators	Strengths	Limitations
National Violent Death Reporting System (NVDRS)	Violent death data from 17 states	Rates of violent deaths including homicide and suicide. Specific mortality rates, such as child maltreatment or child abuse fatalities	Allows examination of detailed information on the incident. It is also possible to identify multiple victim incidents and homicide-suicide occurrences	Not nationally representative
		Rates by different demographic and geographic characteristics	Utilizes linked data from death certificates, coroners/medical examiners, and law enforcement	Focus on fatality misses nonfatal violent events
			Standardized data elements from different sources; uniform relational database for easy queries	
Mortality data	Death data from vital statistics	Mortality rate including homicide and suicide. Allows the calculation of cause specific mortality such as child abuse fatalities	Allows examination of all causes of mortality	Only includes data on deceased
		Spatial analysis and demographic data can be examined	Allows small area analysis (using multiple years as needed)	Coding cause of death may be complex and difficult
			Can be linked with other datasets	Limited to cause of death and demographic data
				Lacks contextual data
Crime Incident Data	Crime incident report from law enforcement agencies	Rates can be calculated for individual crimes such as homicide, aggravated assault, sexual assault, and theft. Data can also be categorized by seriousness of the crime and rates can be calculated	Allow examination of different types of crimes	Less serious crimes are under reported
			Spatial analysis and demographic data can be examined	Data may be biased due to racial stereotyping and neighborhoods
			Has high face validity	Some forms of crimes such as sexual assault are under-reported
				May be affected by changing reporting practices
Discipline Incident Data	Disciplinary incidents, remedial actions, referrals and school characteristics collected by schools and departments of education	Rates of violence related incidents including fighting, weapon possession, homicide, rape, threat, gang related activity, theft, disorderly conduct, and substance possessions	Allows understanding of violence activities at schools	Drop out and frequently truant students may not be represented
		Rates of school dropout, truancy, and truancy conference	Data can be linked with other robust datasets	Inconsistent reporting and definition of data elements
		Demographic characteristics of students	Some neighborhood level data can be obtained for spatial analysis	Lacks contextual data
		Characteristics of schools including accreditation and teacher student ratio		May be affected by changing reporting practices
Juvenile Justice Services	Offers individual level data on youth who are referred to the juvenile justice system	Number and proportion of youth referred to justice systems for violence related activities; including fighting, weapon possessions, rape, threat, gang related activity, theft, disorderly conduct, and substance possessions	Allows close examination of high-risk youth	Data are limited to youth referred to juvenile system
			Data can be linked with other robust datasets	Inconsistent reporting and definition of data elements may bias estimates and trends
			Some neighborhood level data can be obtained for spatial analysis	
Emergency Department (ED) Data	Individual level data on patients treated at the ED for violence related injuries	Rates of assault (including child abuse, homicide and firearm injury), and suicide	Commonly used source of data for measuring and monitoring violence	Not representative of all forms of violence; focus on violence that results in serious or fatal injury. However, not all fatal injuries are captured because some die at the scene and are never taken to the ED
			Can be linked to other databases	
			Allows examination of demographic factors and special analysis	
Ambulance Data	Includes data on violence related traumatic incidents that required ambulance pick up	Rates of assault, fire arm related injuries, rape, fight, stabbing or penetrating injuries	Data can be used to evaluate community efforts and guide policy	Only represents violent events that require immediate medical attention
			Used to estimate rates of violence within a specific community	Typically based on patient report and initial examination; accuracy may be a concern
			Allows spatial analysis on location of the incident and residence of the victim	Inconsistent definition and data collection by different ambulance agencies
			Demographic characteristics can be examined	
Data from the Chief Medical Examiner (CME)	Medical examiner data on violence related mortality	Rates of homicide, including child abuse fatalities, suicide, and firearm related mortality	Provides accurate and in depth information on violence	Data are not made available for timely utilization unless chart reviews are conducted locally
			Can be linked to other databases	
			Accurate; useful for examining trends	
			Lends itself to spatial analysis	
			Good for case studies	
Youth Risk Behavior Surveillance System (YRBSS)	A national survey administered to 9–12 grade students	Provides summary statistics, prevalence and trends at the national, state, and local level	Comprehensive survey on key violence and health issues affecting youth	Lack of data in few states
		Rates of physical fighting, dating violence, sexual violence, weapon possession, threatened with a weapon and physical fight	Reliable self-report data	Participation rates may differ by school in each participating state
		Factors and determinants of youth violence including demographic, life style, and health behaviors	Representative data that can be generalizable (47 states)	Does not include students who miss day of survey administration; may be missing highest-risk youth

### Surveillance Data for Fatal Outcomes

#### National Violent Death Reporting System (NVDRS)

The NVDRS is an active state-based surveillance system designed to collect information on violent deaths including suicide, homicide, unintentional firearm, legal intervention, child maltreatment, and other undetermined injuries (CDC, 2014i). Colorado, Maryland, Michigan, North Carolina and Virginia are among the 32 states that are part of the NVDRS. These data provide accurate information on violence that leads to fatalities as the basis for conducting injury surveillance and for developing and evaluating violence prevention programs. All types of homicides, including variables such as the victim-suspect relationship (e.g., intimate partner, child-parent) are captured. Additionally, since NVDRS includes and links all deaths that occur as part of the same incident, we can identify homicide-suicides and multiple victim incidents. NVDRS also captures legal intervention deaths (individuals who are killed by law enforcement officers and officers who are killed in the line of duty). Data on alcohol and drug involvement based on autopsy results are also collected. The data available for analysis are robust and include the date, time and manner of death, demographic information, and narratives from the coroner or medical examiner and law enforcement officers.

A major benefit of the NVDRS is that it links data from multiple sources, including death certificates, coroner or medical examiner reports, crime incident reports, and crime laboratories (CDC, 2014j).The NVDRS provides unique insight into potential risk factors for both single and multiple victim events and can assist in the development and evaluation of programs and policies designed to prevent deaths from violence. NVDRS data are useful for informing decision makers and program planners about the magnitude, trends, and characteristics of violent deaths in their state or community that can then serve to inform and evaluate prevention efforts. For instance, JHU is drawing upon NVDRS data about alcohol-involved homicides to augment its alcohol policy intervention research, and the VCU maps the data to identify areas with high rates of mortality.

The NVDRS data are also available for query through the Web-based Injury Statistics Query and Reporting System (WISQARS). WISQARS is an online database that can be queried to provide fatal and nonfatal injury, violent death, and cost-of-injury data from a variety of sources (CDC, 2014i). WISQARS is operated by the CDC to provide summary statistics, including frequencies and rates by specified search criteria in the United States. NVDRS WISQARS only reports state-level data. However, smaller geographic level data are available in the Restricted Access Database (RAD) which can be accessed with special request. All of the YVPCs use WISQARS data to determine and monitor violence rates in their respective states and occasionally to evaluate policies (CDC, 2014k).

Despite its strengths, the NVDRS data are not made available for utilization in real time. In most cases, the data are about 3 years old. However, the data provide useful information to examine rates and the impact of implemented prevention approaches.

#### Death Certificate Data

YVPCs use mortality data from their state’s vital registry to monitor youth violence locally. Mortality data are publicly available in most states and can be obtained from the state vital record offices. The type of data available may vary from state to state. Typically, the data provide information on cause of death and the victims’ characteristics.

The death certificate data are helpful in determining and monitoring violence-related mortality rates in any community. Data can be used for small area analysis to guide targeted interventions. Largely, death certificate data are accurate and reliable; however, they only capture the basic demographic characteristics of the deceased. The data can be linked to other data sources using social security number, date of birth and other identifiers for further analysis. Linked datasets allow for the examination of determinants as well as risk and protective factors that are not readily available for researchers (Putnam-Hornstein, Cleves, Licht, & Needell, 2013). Because administrative datasets may not accurately document identifiers, linking data may be challenging. Probabilistic matching (Clark, 2004; Jaro, 1995), a method that uses data across multiple fields to link cases from separate datasets, may be a useful and necessary tool when linking large administrative databases. Nonetheless, obtaining personal identifiers such as social security numbers and date of birth requires permission from vital statistics and health departments. While data that include personal identifiers may be of interest to some researchers, they may not be necessary for stakeholders seeking to develop, plan, implement, and evaluate youth violence prevention programs and policies. YVPCs use local and national death certificate data to evaluate interventions and policies. For instance, the YVPC at Johns Hopkins used the national death certificate data from the National Center for Health Statistics to evaluate firearm laws concerning adolescent suicide rates (Webster, Vernick, Zeoli, & Manganello, 2004).

Moreover, mortality data measure the most severe consequence of violence. However, these data do not provide an overall estimate of the prevalence of violence, which is much more common than fatal injuries. The data only contain basic demographic characteristics and cause of death and do not support the examination of other contextual factors that may be important in examining violence data. Further, death certificate data include a number of primary and secondary variables for cause of death, which make the data difficult to code. The complexity of coding this variable may lead to the misclassification of specific forms of mortality.

#### Uniform Crime Reports (UCR) and Supplemental Homicide Reports (SHR) Data

Another source of data on lethal violence available at the national, state, and local levels is the Federal Bureau of Investigation’s UCR and SHR systems that collect data on murders and non-negligent manslaughters. The SHR provides incident-level data on important factors not available from public health sources including the suspects’ age and relationship to the victim, and the circumstance category (e.g., robbery, drug-related). The UCR and SHR provide data that can be used for smaller geographic areas. The UCR is discussed in great details below.

### Surveillance Data for Nonfatal Outcomes

The YVPCs use several locally and nationally available surveillance data sources to monitor youth violence and evaluate youth violence prevention efforts. While most of the data are publicly available, identifiable data are often restricted. However, YVPCs have established local collaborations and instituted specific agreements that allow them to utilize these data. This section describes the most widely available data sources that are used by YVPCs.

#### Crime Incident Data

All of the YVPCs use crime incident data to monitor youth violence rates and measure the impact of their interventions. Crime incident data are the standard measure of crime used by law enforcement, media, citizens and academics to characterize the level of violent and non-violent crime in a geographic location. Local data can be collected from law enforcement agencies including crime incident and arrest reports. These data generally consist of a number of characteristics of the crime incident, including date, time, location, and classification of the nature of the criminal incident. The YVPCs use the standard definitions and mechanism for recording and reporting crime incidents provided by the Federal Bureau of Investigation (FBI) Uniform Crime Reports (UCR; FBI, 2014). These classifications include definitions of violent crimes (e.g., murder, non-negligent manslaughter, aggravated assault, aggravated battery, criminal sexual assault, and robbery) as well as other, non-violent crimes (e.g., theft, burglary, and vandalism). Crimes may also be categorized as index (i.e., more serious offenses) versus non-index crimes. A hierarchy among crime categories exists so incidents that, for example, involve an assault, a robbery and rape are categorized based on the most severe offense, with the level of severity descending from rape, to robbery, to assault.

Crime incident data have a number of advantages as indicators of community violence in a surveillance system. First, these data generally have very high face validity due to their frequent use to report crime statistics in the media, by law enforcement, and in academic journals. Second, reporting systems have been in place for decades, thus providing useful opportunities to examine trends in violent crime over time. Third, with the increasing collection of detailed incident information, including geocoded location, these data can be easily aggregated to provide statistics for a defined geographic area. This allows for relatively easy integration with other sources of data (e.g., census, community surveys, or other location-based data). The ability to use longitudinal geocoded data on incidents of violent crime and arrests using standard definitions is incredibly valuable for describing variations in youth violence over time and place, providing the capability to evaluate the efficacy of neighborhood or place-based interventions.

The Johns Hopkins YVPC has used Baltimore’s Police Department data on murders, non-negligent manslaughters, and nonfatal shootings as well as arrests for weapon and drug violations to evaluate youth violence prevention interventions such as Safe Streets (based on the Cure Violence model) and law enforcement programs focused on violent gun offenders (Webster, Whitehill, Vernick, & Curriero, 2012). The Hopkins YVPC has also used local police data to examine the relationship between violent crime incidents, arrests for alcohol-related crime, and the location of alcohol outlets to advance policies to curtail problem alcohol outlets (Jennings et al., 2014). The Colorado YVPC utilized Denver’s violent crime data to select intervention and control neighborhoods for their study. Neighborhoods that ranked in the upper one-third of violent crime for Denver were deemed eligible to participate. Neighborhood level violent crime data are also being used to evaluate outcomes.

Although incident data can sometimes be difficult to obtain and have not always been released in a timely fashion, these data are increasingly accessible through government-sponsored websites that make crime maps and the raw incident data routinely available. For example, the City of Chicago hosts the *Chicago Data Portal* (www.data.cityofchicago.org), a data repository that contains a wide range of data related to the city (City of Chicago, 2014). Included on this site are all crime incidents from 2000 to the present. These data are updated daily and can be accessed and downloaded without restriction. These data were central to the process used by the Chicago YVPC to select their target and comparison communities. The Chicago YVPC continues to use crime incident data to monitor crime in the target community and to evaluate the impact of the YVPC over time.

In addition, researchers from the Chicago YVPC have explored the use of crime incident data as a form of sentinel surveillance (Henry et al., 2014). Henry and colleagues used data on incidents of disorderly conduct, vandalism, and weapons violations to predict future increases in neighborhood-level violent crimes. The study suggested the potential use of crime incident data to anticipate neighborhoods at risk for future increases in violence and to target prevention strategies accordingly.

Despite the strengths of crime incident data, there are some limitations that must be considered when using these data as part of a surveillance system. As with any single component of surveillance, there are biases inherent in incident data. The primary issue to consider is that for most types of crime, not all cases are reported to the police and recorded in the database (e.g., Skogan, 1984). Typically, more severe crimes (e.g., homicide, violent crimes that involve major injuries, major theft) are more likely to be reported and recorded than less severe crimes; however, underreporting is a particular problem for sexual assault. Data from the 2013 National Crime Victimization Survey rates of reporting victimizations to police were 36.1 % for property crimes, 45.6 % for violent crimes, and 61.0 % for serious violent crimes (Truman & Langton, 2014). Of note, the reporting rate for rape and sexual assault was only 34.8 %.

Within challenged communities that are the focus of the YVPCs, there may be several competing biases in the rates for crime incident and arrest data. Within inner city, low income neighborhoods or other communities that are frequently patrolled by law enforcement officers, individuals may be more likely to be cited or reported for minor crimes than incidents occurring in other areas. Further, racial stereotyping in predominantly minority neighborhoods could also lead to disproportionate arrest rates. While policing patterns may differentially raise incident and arrest rates, distrust of law enforcement, perceptions of ineffective policing, and social norms against reporting to law enforcement can serve to lower rates in these areas. In the context of using incident data to evaluate community-level interventions, changing reporting practices, whether intended or not, may alter measured incidence rates when actual crime rates have not changed. For example, efforts to enhance block club participation such as neighborhood walking or gardening clubs and neighborhood watch may empower residents to report crimes that previously went unreported.

#### Discipline Incident Data

All of the YVPCs use disciplinary incident data to measure outcomes and monitor youth violence in schools. The YVPCs collaborate with schools and state and county departments of education to obtain data on disciplinary incidents, remedial actions, referrals and school characteristics. The type of data available varies by YVPC but typically includes school and student characteristics.

Data from schools provide useful information to understand school-based violence. For instance, the YVPC at VCU monitors trends of youth violence rates including weapon possession, fights, bullying, truancy, and drop outs (Masho & Bishop, 2014a). Furthermore, school disciplinary data are linked to student survey data to measure individual level outcomes. UNC uses school disciplinary data to monitor acts of youth violence committed during the school year. Data related to weapon possession, possession of controlled substances and alcoholic beverages, assault, and sexual assault are examined. The Chicago YVPC is incorporating school disciplinary data into the evaluation of school-based interventions that are included in their comprehensive violence prevention strategy. Disciplinary data at the school and grade (within school) level allow the evaluation team to assess changes in disciplinary incidents and to compare schools in the intervention community to those in the comparison community as well as to all Chicago Public Schools. By linking schools to neighborhoods, covariates in these models include community-level as well as school-level factors.

Despite these benefits, disciplinary incident data have some limitations. First, the data are limited to incidents that occurred on school grounds and do not provide accurate estimates of violence outside school. Second, the data are limited to students who attend school. Drop out and chronically tardy students who are at a higher risk of violence may not be adequately represented. Third, data are limited to certain demographic and school characteristics and do not allow for the examination of risk and protective factors. Fourth, the uniformity of the data may vary by school culture and school-specific initiatives. For instance, a school with a bullying prevention program may be more diligent in collecting violence related data which may indicate changes in bullying. Therefore, a clear understanding of contextual issues affecting data collection is important.

#### Juvenile Justice Services Data

Few of the YVPCs use juvenile justice services data to monitor the impact of their program. The type of data available varies by YVPC site; however, data typically include individual-level data on youth who were referred to the juvenile justice system or have been formally processed or sentenced by juvenile court. Although the data collected may differ by locality, demographic information, and type of offense, sentence and referral services provided are consistently documented across sites. The Office of Juvenile Justice and Delinquency Prevention (OJJDP) has a website that offers a collection of national juvenile justice datasets (OJJDP, 2014).

The juvenile justice services data allow a close examination of youth who are at increased risk of behavioral problems. UNC uses juvenile justice services data to examine juvenile arrests, delinquent acts and complaints against juveniles. VCU has tracked juvenile justice services data since 2003 to monitor violence-related offenses including homicide, assault, sexual assault, weapons and bombs, robbery and kidnapping. The data are also being used to monitor trends and changes in the community. For instance, a recent analysis of the data in Richmond, VA found a gradual increase in the rate of females referred to juvenile justice services (Masho & Bishop, 2014b). This information provided the impetus to monitor and understand gender differences in youth violence. Furthermore, the data are being used to assess the impact of targeted interventions among these high risk youths at VCU.

Although the juvenile justice services data offer several advantages, they have some limitations. Juvenile justice system data are administrative data collected on youth who have been identified and referred to the juvenile justice system. The introduction of initiatives targeting youth may lead to over- or under-reporting that may inflate or deflate data. For example, in Richmond, VA, a truancy initiative resulted in increased reporting of truancy making other forms of violence seem lower (unpublished data).

#### Emergency Department (ED) Data

ED data are being used by all of the YVPCs. The type of data collected may vary by site. However, they typically include patients’ demographic characteristics, diagnosis and procedure codes. To determine injury groupings, the recommended framework for presenting injury mortality data, the International Classification of Diseases, Ninth Revision (ICD-9) provided by CDC is being used to summarize the data. External Cause of Injury Codes (E-codes) are used to determine injury groupings. These groupings include presence of injury, external injury, motor vehicle crash, assault, child abuse, suicide and firearm injury (CDC, 2014o). The YVPC at VCU has partnered with its tertiary trauma center and collects data to assess the burden of youth violence locally (Masho & Bishop, 2014c). The data are being used to monitor trends in youth violence in Richmond City and identify populations that are disproportionately affected by the problem. The data are also being used to estimate rates of ED visits attributed to violence and examine the impact of local interventions.

ED data are also available nationally from the National Electronic Injury Surveillance System—All Injury Program (NEISS-AIP). The system is operated by the U.S. Consumer Product Safety Commission with CDC’s National Center for Injury Prevention and Control (NCIPC). These data incorporate reports with national estimates of nonfatal injuries treated in U.S. hospital emergency departments broken down by intent (physical assault, sexual assault, legal intervention, self-harm, unintentional) and cause and mechanism (e.g., firearm, cut or pierce). Using these same data, the CDC’s NCIPC generates the leading cause of nonfatal injury rankings for the top 10 causes of nonfatal injuries treated in EDs by the age and sex of the injured patient, intent of injury, and disposition when released from the ED. The YVPCs use these data to compare local rates with national rates. Although the NEISS-AIP offers similar information, the data usually lag by a few years. Local ED data offer more timely and robust information that can provide immediate local benefit.

ED data are one of the most commonly used data sources to measure violence, and include demographic and medical information. However, ED data capture only victimized youth who seek medical attention—typically more severe violence. These data exclude the majority of youth involved with violent episodes who are not injured as well as those who are injured but do not require medical attention.

#### Ambulance Pick-Up

VCU uses ambulance data to assess the trends of more severe violence in the community. Similar to ED data, ambulance data are used to estimate rates of violence in the community. These data include information on the demographic characteristics of the victim and call disposition. The call disposition information is used to determine violent incidents. Events where an assault occurred, such as rape, a fight or brawl, shooting, or stabbing are categorized as violent injury-related events.

The data are used to evaluate community-wide efforts and guide policies. For instance, a report by Masho et al. (2014) utilized ambulance data to examine the impact of restricted alcoholic beverage licenses on rates of ambulance pickup in areas where violence rates were high. For example, a report of the surveillance data depicting higher rates of violence surrounding grocery stores selling less expensive alcoholic beverages informed the effort made by a local civic organization to place restrictions on the sale of single-serve alcoholic beverages.

Despite their strengths, ambulance data have a number of limitations. Similar to ED data, ambulance data only represent injuries that require immediate medical attention. Furthermore, dispositions are usually based on patient report and initial examination and may be inaccurate. It is also important to note that most cities have more than one ambulance provider, and data collected by multiple companies may lack uniformity.

#### Youth Risk Behavior Surveillance System (YRBSS)

The YRBSS is a national survey administered by the CDC biennially (CDC, 2014f). All of the YVPCs use YRBSS data to monitor youth violence risk factors. The YRBSS began in 1991 to systematically collect data on a wide range of adolescent behaviors, including violence related activities. The school-based survey, which covers grades 9 through 12, is anonymous and cross-sectional. A middle school YRBSS is also established, but is currently used by only 18 states. Five states where the YVPCs are located (Colorado, Maryland, Michigan, North Carolina, and Virginia) collect data on middle schools. However, the data may not be representative of specific localities to evaluate programs.

YVPCs rely on the YRBSS to measure current behavior and monitor trends over time at the state level. YVPC researchers often use YRBSS measures to ensure that their measures of youth violence and risks are comparable to data available in many cities and states. Although 47 states implement the survey, participation rates by school may differ by states. Decisions to participate in the YRBSS may be mandated by government officials, or left to the discretion of school administrators. Thus, a significant proportion of schools may opt not to participate, limiting its generalizability. For example, in Colorado, state level data are available dating back to 1995, but Denver public schools did not participate in the YRBSS until 2007—therefore, arguably the highest risk population in Colorado was not represented until then. Additionally, YRBSS data are based on students who were present or available on the day of the survey; thus, the highest risk students may be truant and not represented. The YRBSS website has an extensive explanation of study design, and multiple reports and manuscripts have been published on the most recent trends in adolescent behavior (CDC, 2014m).

The YRBSS data are publicly available and can be downloaded for further analysis. Additionally, summary statistics from the YRBSS data can be accessed from the Youth Online portal (CDC, 2014n). The portal provides statistics and trends at the national, state, and local levels, depending on the availability of weighted data for a particular geographic location. For instance, weighted data are available for Colorado for years 2005, 2009, and 2011. The data revealed that there has been a decrease in physical fighting for the first time in a decade. Comparing data from 2009 to 2011, physical fighting decreased from 32 to 24.9 % *p* < 0.001. Further analysis showed that the differences are accounted for by changes in fighting among males: 42–30.3 %, *p* < 0.001 and are not due to change in female fighting, which also declined from 21.8 to 18.2 %, (*p* = 0.16) (CDC, 2014l).

The Colorado YVPC incorporates several YRBSS violence related questions into their community survey. This allows for the comparison of state level changes for physical fighting (as an example), over time to intervention community changes to help determine whether their intensive community level efforts to decrease youth violence can be attributed to the intervention.

### Census Data

Although census data are not designed for surveillance purposes, the YVPCs use census data to calculate rates of violence in the community. The United States Census Bureau has been collecting counts of the population of the United States since the first decennial census in 1790 (Gauthier, 2002). These population counts serve a critical role as the denominator in calculating youth violence rates by the YVPCs. In addition to population totals, information collected by the Census Bureau provides data to be incorporated into youth violence surveillance systems. Examples of these variables include income, education, employment, and housing tenure. These variables serve as important markers of risk factors at various levels of the community geography (e.g., county, zip code, tract, block group). For example, Tolan, Gorman-Smith, and Henry (2003) used census data to create two measures of community structural characteristics: concentrated poverty (rates of poverty, unemployment, female-headed households, and owner-occupied housing) and ethnic heterogeneity (number of ethnic groups and number of languages) in a developmental, ecological model predicting youth violence. All of the YVPCs used neighborhood demographic characteristics in the process for selecting the target communities and identifying demographically-similar comparison communities. For example, the YVPC at VCU compared census tract level demographic data from the American Community Survey (ACS) to select intervention and comparison communities. Additionally, these data are used as a denominator when calculating rates of youth violence in the community.

Once collected from a sample of the population as the “long form” in the decennial census, many of these additional demographic characteristics are now included in the ACS (U.S. Census Bureau, 2014). Beginning, in 2005, the ACS has surveyed a sample of the population regularly. These data form annual estimates of population characteristics at varying levels of geography. Single-year estimates are available for population units greater than 60,000; 3-year estimates for greater than 20,000; and 5-year estimates for population units as small as the block group.

Data from the decennial censuses, ACS, and other data collected by the Census Bureau are available for download at American Fact Finder (http://factfinder2.census.gov). As with one- and three-year estimates for the ACS, many of the other datasets are available only for larger population areas. Despite this limitation, the YVPCs use the data as a denominator when estimating rates of violence in a defined geographic area.

## Key Consideration for Data Analysis

In this section we will highlight some of the key issues that YVPCs are considering when undergoing integration and analysis of the types of surveillance data described above. For extended coverage of the process of mapping and analyzing crime data, one recommended source is a textbook by Chainey and Ratcliffe (2005).

The data used by the YVPCs include a mix of sources at varying levels of aggregation. At the lowest level of aggregation are individuals such as crime victims, students, or patients. Other data are aggregated to varying geographic units ranging from relatively small geographic areas such as census block groups to increasingly larger regions such as school attendance zone, county, or state. A challenge presented when attempting to relate data from multiple sources is that the data are often aggregated to different, overlapping geographic spaces. For example, relevant surveillance data may be available at the level of the police beat (Chicago), zip code, and census tract (Virginia), none of which maps onto the same geographic space.

Surveillance data may often be presented using maps to display correlations between multiple factors in the geographic landscape. For instance, the YVPC at VCU publishes factsheets to monitor crime rates in Richmond City using maps (Masho & Bishop, 2014b). A common use of maps is to visually present crime density, an approach often referred to as hotspot analysis. This analysis allows the researchers to present areas of relatively high crime density and relate them to other characteristics of the areas in which the crimes occur. For example, a consistent finding in the literature is the relation between retail alcohol outlets and violent crime. In addition to quantitative analyses demonstrating these effects, Lipton and colleagues used maps to highlight this relationship (Lipton et al., 2013).

Even when considering individual-level data, YVPCs are generally focused on understanding youth violence in the context of a specific geographic area. As such, the modeling of individual cases within geographic or administrative units is an important component of analytic models of these data. A number of texts have addressed general issues of multilevel models (i.e., models in which individuals are clustered within higher-level units), including Raudenbush and Bryk (2002) and Singer and Willett (2003).

Similar to multilevel data, when dealing with geospatial data assumptions of independence are often violated. In the case of geospatial data, neighboring areas are likely to influence each other in ways that likely violate assumptions of traditional analytic methods used in the social sciences. The dependencies between neighboring areas are modeled as a function of proximity to neighboring areas using methods such as geographically weighted regression (Fotheringham, Brunsdon, & Charlton, 2002), two-stage least squares regression (Kelejian & Prucha, 1998), and Bayesian conditional autoregressive models (Besag, York, & Mollié, 1991). As noted above, details of these methods are available in standard text books.

## Conclusion

Surveillance data are vital for monitoring and describing youth violence, identifying places and times, and evaluating the impact of interventions implemented at the neighborhood, city, state or national levels. We have described data sources, outcome indicators and considerations for analytic approaches as used by the YVPCs. Each source of surveillance data has strengths and limitations. Most sources of surveillance data have information on victims’ characteristics, but few have information on perpetrators of youth violence, their motivations or relationships to victims, or other aspects relevant to circumstances surrounding incidents of violence.

YVPCs accurately track fatalities using death certificates, medical examiner records, and police reports. Fatalities are rare events, and most youth violence does not lead to fatal outcomes. The YVPCs also monitor nonlethal acts of youth violence using hospital emergency department data, ambulance calls, police incident reports, and school disciplinary records. Changes in reporting practices with these surveillance data sources are often not systematically documented which, if not accounted for, can bias comparisons across place and time. Combining contextual data from the Census or other government sources regarding the population at risk or factors relevant to jurisdictions being studied (e.g., alcohol outlets, vacant buildings) greatly expands the utility of surveillance data for monitoring and studying youth violence provided the surveillance data include information on the location of the incidents of those involved.

In summary, youth violence surveillance data provide a great opportunity to examine community-level outcome indicators that can be used to monitor and evaluate youth violence prevention programs. Our ability to understand the causes of youth violence and determine what interventions are most effective in preventing it depend on systems for the standardized recording of violence incidents involving youth within defined populations or settings. Efforts to improve the quality of surveillance data and analytic approaches to analyzing those data, therefore, will be of great value to the field of youth violence prevention.
